# Pulse Waveform Changes During Vasopressor Therapy Assessed Using Remote Photoplethysmography: A Case Series

**DOI:** 10.3390/jcm15031118

**Published:** 2026-01-30

**Authors:** Mara Klibus, Viktorija Serova, Uldis Rubins, Zbignevs Marcinkevics, Andris Grabovskis, Olegs Sabelnikovs

**Affiliations:** 1 Department of Anaesthesiology, Intensive Care and Clinical Simulations, Riga Stradins University, LV-1007 Riga, Latvia; 2 Department of Intensive Care, Pauls Stradins Clinical University Hospital, LV-1002 Riga, Latvia; 3 Faculty of Science and Technology, University of Latvia, LV-1586 Riga, Latvia; 4 Faculty of Medicine and Life Sciences, University of Latvia, LV-1586 Riga, Latvia

**Keywords:** septic shock, vasopressors, norepinephrine, remote photoplethysmography, microcirculation, perfusion index

## Abstract

**Background/Objectives**: Septic shock involves severe circulatory and microcirculatory dysfunction and often requires vasopressors to maintain adequate mean arterial pressure (MAP). Conventional monitoring mainly reflects macrocirculation and may not capture changes in vascular tone or microcirculation. Remote photoplethysmography (rPPG) is a contactless optical method that analyzes peripheral pulse waveforms and may offer additional physiological insight during vasopressor therapy. The aim of this study was to assess the feasibility of rPPG for detecting pulse waveform changes associated with norepinephrine administration in septic shock. **Methods:** Prospective case series included three adult patients (*n* = 3) with septic shock admitted to the intensive care unit at Pauls Stradins Clinical University Hospital, Riga, Latvia. All patients received standard sepsis treatment, including fluid resuscitation and titrated norepinephrine to maintain MAP ≥ 65 mmHg. Continuous invasive arterial pressure monitoring was performed alongside rPPG signal acquisition from the palmar skin surface under controlled lighting. From averaged rPPG waveforms, perfusion index (PI), dicrotic notch amplitude (c-wave), and diastolic wave amplitude (d-wave) were extracted. Correlations between norepinephrine dose, MAP, and rPPG parameters were explored. **Results:** Increasing norepinephrine doses were associated with higher MAP and PI in all patients. Dicrotic notch and diastolic wave amplitude decreased consistently. These changes occurred alongside macrocirculatory stabilization and are consistent with increased vascular tone and altered arterial compliance. **Conclusions:** rPPG demonstrated feasibility for detecting pulse waveform changes during norepinephrine therapy in septic shock; however, larger controlled studies are required for validation.

## 1. Introduction

Sepsis remains a major challenge in intensive care medicine and is defined as a dysregulated host response to infection leading to life-threatening organ dysfunction. When sepsis progresses to septic shock, patients develop profound circulatory, cellular, and metabolic abnormalities that require vasopressor therapy to maintain adequate perfusion pressure and are associated with high mortality rates, often exceeding 40% despite advances in critical care management [1,2].

According to current definitions, septic shock is characterized by the need for vasopressors to maintain a mean arterial pressure (MAP) of at least 65 mmHg in the presence of elevated serum lactate levels despite adequate fluid resuscitation [2]. While MAP remains a cornerstone of hemodynamic management, it primarily reflects macrocirculatory status and does not reliably capture alterations at the microcirculatory level. Importantly, restoration of systemic blood pressure does not necessarily translate into normalization of tissue perfusion, a phenomenon described as loss of hemodynamic coherence (i.e., a mismatch between macrocirculatory stabilization and persistent microcirculatory dysfunction) [3,4,5,6].

Microcirculatory dysfunction plays a central role in the pathophysiology of septic shock. Inflammatory mediator release, endothelial injury, glycocalyx degradation, coagulation abnormalities, and impaired vasoregulation lead to heterogeneous capillary blood flow and impaired oxygen extraction at the tissue level. These microvascular disturbances may persist even after apparent stabilization of macrocirculatory variables, contributing to ongoing organ dysfunction and adverse outcomes [3,4,5].

Current bedside methods used to assess tissue perfusion, such as serum lactate concentration and capillary refill time, have important limitations. Lactate is influenced by factors beyond hypoperfusion, including altered cellular metabolism, while capillary refill time is subjective and operator dependent. Advanced techniques for direct microcirculatory assessment, including sublingual video microscopy and near-infrared spectroscopy, provide more detailed information but are limited by technical complexity, cost, and restricted availability in routine clinical practice [4,5,6]. As a result, there is an unmet need for simple, non-invasive tools capable of providing real-time insight into peripheral vascular and microcirculatory changes during septic shock treatment.

Photoplethysmography (PPG) is a non-invasive optical technique that detects blood volume changes in the microvascular bed and has been investigated for clinical applications for more than seven decades [7,8]. In contemporary clinical practice, PPG is widely used in pulse oximetry and has been increasingly explored for assessment of tissue perfusion, vascular tone, and pulse waveform morphology. Parameters derived from PPG, including perfusion index, waveform variability, and morphological features, have demonstrated sensitivity to changes in vascular resistance, arterial compliance, and sympathetic tone, making PPG particularly relevant in conditions such as septic shock and during vasopressor therapy [7,9,10,11].

Photoplethysmography (PPG) has been widely studied as a noninvasive approach for cuffless blood pressure estimation. Prior investigations have shown that temporal, amplitude-based, and morphological features of the PPG waveform—as well as its first and second derivatives—are associated with systolic and diastolic blood pressure and can be incorporated into predictive models. More recent studies have evaluated the performance, robustness, and methodological limitations of PPG-based blood pressure estimation, emphasizing the importance of signal quality, calibration strategies, and standardized validation protocols [12,13,14].

Although these findings demonstrate the strong sensitivity of PPG signals to changes in arterial pressure and vascular properties, they also indicate that PPG reflects integrated macrovascular and microvascular dynamics rather than isolated blood pressure values alone. This characteristic reinforces the value of analyzing PPG waveform behavior to characterize peripheral vascular responses during vasopressor therapy.

Photoplethysmography (PPG) is a noninvasive optical technique widely used to monitor pulsatile blood volume changes and assess cardiovascular and perfusion dynamics, typically through contact-based sensors attached directly to the skin. Although conventional PPG is well established, probe pressure, local compression, motion artifacts, and compromised skin integrity may affect signal quality and patient safety, particularly in critically ill patients with edema, vasoconstriction, fragile skin, or frequent handling. Remote photoplethysmography (rPPG) extends this technology by enabling contactless, imaging-based acquisition of PPG signals using standard video cameras and ambient light, extracting physiological information from subtle color fluctuations of exposed skin regions. This approach offers several potential advantages in the intensive care setting, including reduced risk of skin injury and infection, avoidance of sensor-related artifacts, and the possibility of continuous monitoring without direct patient contact. While several studies in sepsis have used contact-based PPG to assess perfusion or waveform features, recent work has demonstrated the feasibility of rPPG primarily in controlled environments, and evidence for its physiological validity and clinical applicability in the intensive care unit—particularly during active hemodynamic interventions such as vasopressor titration—remains limited [15,16].

Vasopressor therapy, particularly with norepinephrine, is a cornerstone of septic shock management. While norepinephrine effectively restores MAP by increasing systemic vascular resistance, its effects on peripheral perfusion and microcirculatory flow are complex and may vary between patients. Conventional monitoring may therefore fail to capture important changes in vascular tone and pulse wave dynamics induced by vasopressor titration [9,17,18,19].

The aim of this case series was to explore the feasibility of using rPPG to detect pulse waveform changes during norepinephrine therapy in patients with septic shock. Specifically, we sought to evaluate changes in rPPG-derived perfusion index and waveform morphology in parallel with macrocirculatory variables, and to assess whether rPPG may provide complementary physiological information during vasopressor treatment in the intensive care setting.

## 2. Materials and Methods

### 2.1. Study Design and Setting

This prospective observational case series was conducted in the intensive care unit (ICU) of Pauls Stradiņš Clinical University Hospital, Riga, Latvia. The study aimed to explore the feasibility of remote photoplethysmography (rPPG) for detecting vascular waveform changes during vasopressor therapy in patients with septic shock.

The study was performed in accordance with the Declaration of Helsinki. Ethical approval, including an approved extension, was obtained from the local institutional ethics committee, and informed consent was obtained from patients’ legally authorized representatives prior to inclusion. The study was approved by the Ethics Committee of Riga Stradiņš University (approval number PĒK-22-2/299/2021).

### 2.2. Patient Selection

Three adult patients (≥18 years) diagnosed with septic shock were consecutively included during a predefined recruitment period (June–August 2025). Septic shock was defined according to the Sepsis-3 criteria, including the requirement for vasopressor therapy to maintain a mean arterial pressure (MAP) ≥65 mmHg despite adequate fluid resuscitation.

All patients were admitted to the ICU for management of severe infection-related organ dysfunction and required invasive mechanical ventilation at the time of data acquisition.

Patients were consecutively included based on predefined eligibility criteria, without additional selection based on hemodynamic severity or other clinical characteristics. In all three cases, rPPG recordings were initiated within the first 3 h after ICU admission during the early stabilization phase, while norepinephrine doses were actively titrated to achieve the target mean arterial pressure, allowing temporal assessment of pulse waveform changes in relation to vasopressor adjustments.

### 2.3. Inclusion Criteria

- Age ≥ 18 years;
- Diagnosis of septic shock;
- Presence of an invasive arterial catheter for continuous blood pressure monitoring;
- Requirement for norepinephrine infusion;
- Invasive mechanical ventilation.

### 2.4. Exclusion Criteria

- Atrial fibrillation or significant cardiac arrhythmias;
- Active major bleeding during current hospitalization;
- Severe neurological injury affecting autonomic regulation;
- Inability to obtain informed consent from a legal representative;
- Patients receiving maximal vasopressor doses with imminent risk of death.

### 2.5. Clinical Protocol

All patients were treated according to the current Surviving Sepsis Campaign guidelines. Standard management included early broad-spectrum antimicrobial therapy, fluid resuscitation with balanced crystalloids (30 mL/kg administered within the first 3 h), and titrated norepinephrine infusion to maintain a target MAP ≥65 mmHg.

Continuous invasive arterial blood pressure monitoring was performed via a radial arterial catheter connected to a bedside monitoring system (Philips MX750, Boblingen GmbH, Germany). Serum lactate levels and other routine laboratory parameters were monitored as part of standard ICU care.

**Figure 1:**
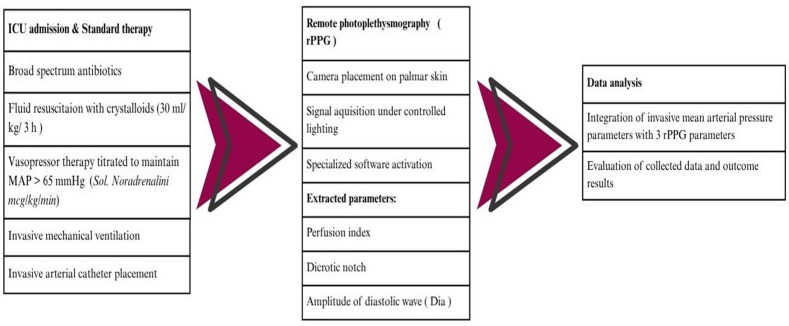
Treatment and measurement protocols.

Patients were sedated and mechanically ventilated using standard ICU sedation protocols. Sedative and analgesic dosing was recorded during the observation period (Figure 1).

### 2.6. Remote PPG Acquisition

**Figure 2:**
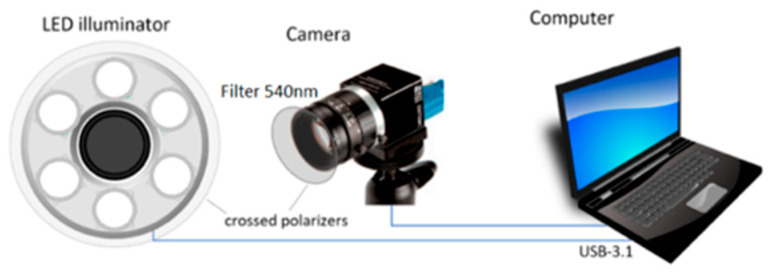
The remote PPG system for signal acquisition from palms Reproduced from [20], © 2024 IEEE.

The prototype remote PPG device (Figure 2) comprises an illumination system—a set of 6 high-power visible-spectrum LEDs, (LumiLed LXZ1-PX01, CWL = 556 nm, FWHM = 150 nm) and a monochromatic camera (Ximea MQ003MG-CM, ADC 12-bits, resolution 648 × 488), equipped with a low-distortion objective and bandpass filter glass ((CWL) = 540 nm, (FWHM) = 10 nm). Orthogonally oriented polarizers were installed in the front of the illuminator and camera to eliminate skin glare. The system was placed on a mobile stand. To achieve a uniform illumination of an adult palm (20 × 15 cm field of view), the device was positioned approximately 35 cm from the subject’s palm.

Remote photoplethysmography signals were acquired non-invasively from the palmar skin surface under controlled ambient lighting conditions. Recordings were performed within the first 3 h after ICU admission during the early hemodynamic stabilization phase, while norepinephrine was actively titrated to achieve the target mean arterial pressure. Video recordings were obtained while patients were in a supine position, and precautions were taken to minimize motion artifacts during data acquisition.

For each patient, rPPG data were analyzed over the entire selected recording period during the stabilization phase, corresponding to approximately 3–4 min of continuous signal acquisition. At typical heart rates observed in our cohort, this represents not less than approximately 180 consecutive pulse cycles per recording segment.

Individual cardiac cycles were segmented and ensemble-averaged to improve the signal-to-noise ratio prior to feature extraction.

### 2.7. Signal Processing and Feature Extraction

Recorded videos were processed offline using MATLAB (version R2020a, MathWorks, Natick, MA, USA). The main steps of signal processing were as follows: (1) the region of interest (RoI) chosen on the palm image to obtain a spatially averaged PPG signal; (2) the cardiac-related PPG signal was obtained by applying a band-pass filtered signal (3rd order Butterworth filter, 0.6–6 Hz); and (3) the segmentation of the PPG signal into individual single-period (sPPG) signals, related to individual heartbeats. The amplitude of each sPPG was normalized within the interval [0–1], the pulse cycles were normalized to [0–1 s], and all systolic peak positions were normalized to 0.25 s by linear stretching of the sPPG signal. (4) Finally, ensemble-averaging of sPPG signals (3 min time window) were performed to increase the signal-to-noise ratio [20].

The quality of the rPPG signal is significantly dependent on perfusion but is also degraded by fluctuations in ambient light and patient palm movements. To evaluate this, the signal-to-noise ratio can be expressed as follows:

$$SNR=10log_{10}\frac{\sum_{f=0.6}^{f=6}\Pi (f)|F(f){|}^{2}}{\sum_{f=0.6}^{f=6}(1-\Pi (f))|F(f){|}^{2}}$$

where *F* is the Fourier spectrum of rPPG signal, taken in a frequency range of 0.6 *< f <* 6 Hz, where *f*_0_ is a dominant frequency of heartbeats. Π(f) is a binary function defined as

$$\Pi \left(f\right)=\left\{\begin{array}{c} 1\;f_{0}-0.1<f<f_{0}+0.1\\ 0\;otherwise \end{array}\right.$$

The following rPPG-derived parameters were extracted:

− Perfusion index (PI), calculated as the ratio of the pulsatile (AC) component to the non-pulsatile (DC) component of the PPG signal, reflecting relative peripheral perfusion and signal amplitude.
− Dicrotic notch amplitude (c-wave), which corresponds to the secondary systolic peak in the second derivative of the PPG waveform and is associated with arterial compliance and wave reflection.
− Diastolic wave amplitude (d-wave), which represents the subsequent negative deflection following the c-wave and has been linked to vascular tone and pulse wave dynamics.

**Figure 3:**
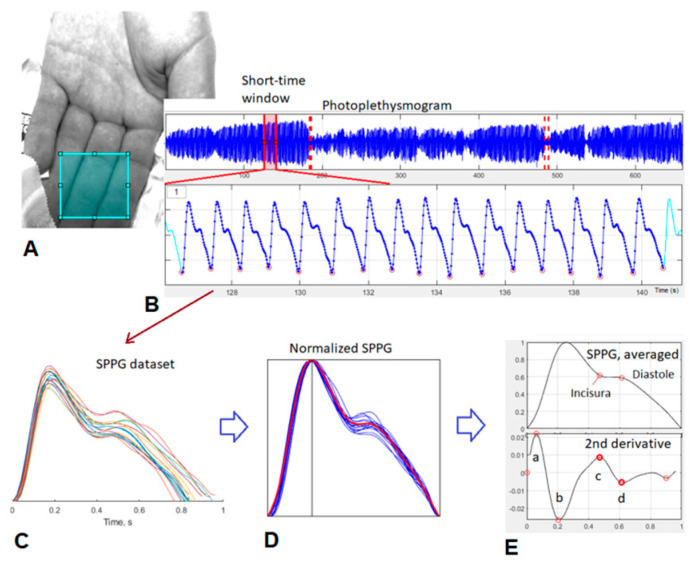
Typical processing pipeline for subspace photoplethysmography (sPPG) signal analysis, adapted from contactless photoplethysmography. The PPG signal is extracted from a manually selected region of interest (ROI) on the palm image (blue rectangle) (**A**). A 3-min time window (red markers) is selected to obtain the cardiac-related sPPG dataset (**B**). Single-period sPPG waveforms are computed (**C**) and normalized to produce the ensemble-averaged sPPG waveform (**D**). The 2nd derivative waveform and inflection points “a,b,c,d” are found and waveform derived parameters are calculated (**E**). Reproduced from [20], © 2024 IEEE.

Second-derivative analysis (acceleration photoplethysmography, i.e., the second derivative of the PPG waveform to enhance morphological features) was applied to further characterize waveform morphology and identify waveform components (Figure 3).

### 2.8. Data Collection

Demographic data, primary diagnosis, Sequential Organ Failure Assessment (SOFA) score, norepinephrine dose, MAP, lactate levels, and rPPG-derived parameters were recorded during the observation period. Changes in rPPG parameters were evaluated in parallel with norepinephrine dose adjustments and corresponding macrocirculatory responses.

### 2.9. Statistical Analysis

Given the exploratory nature of this case series and the small sample size, analyses were primarily descriptive. Associations between norepinephrine dose, mean arterial pressure (MAP), and rPPG-derived parameters were explored using correlation analysis within individual patients.

Pearson’s correlation coefficient was used when data satisfied normality assumptions; otherwise, Spearman’s rank correlation coefficient was applied as a nonparametric alternative. Results are presented as correlation coefficients and percentage changes relative to baseline values. No adjustments were made for multiple comparisons. Statistical analyses were performed using MATLAB software.

## 3. Results

### 3.1. Patient Characteristics

A total of three (n = 3) patients were included in this case series. The SOFA scores ranged from 8 to 13, reflecting significant disease severity on ICU admission.

In all three cases, rPPG recordings were initiated within the first 3 h after ICU admission during the early stabilization phase, while norepinephrine doses were actively titrated to achieve the target mean arterial pressure, allowing temporal assessment of pulse waveform changes in relation to vasopressor adjustments.

The primary diagnoses leading to ICU admission were pneumonia with acute respiratory distress syndrome (ARDS) (n = 1), secondary peritonitis (n = 1), and pleural empyema 195 with pneumonia (n = 1). Patients required norepinephrine infusion at doses ranging from 0.02 to 0.11 µg/kg/min to maintain adequate mean arterial pressure (Table 1).

### 3.2. Case Series Description

Case 1

A 49-year-old female patient was admitted to the hospital with complaints of progressive shortness of breath and cough, accompanied by worsening respiratory failure. The patient had no known chronic medical conditions. On admission, oxygen saturation was 86% despite high-flow oxygen therapy via a face mask.

Chest computed tomography revealed bilateral pneumonia with findings consistent with acute respiratory distress syndrome (ARDS). Urine antigen testing was positive for Legionella pneumophila. Antibacterial therapy with ciprofloxacin was initiated.

Due to progression of respiratory failure, the patient was transferred to the intensive care unit (ICU), with a SOFA score of 8. In view of respiratory compromise and impaired neurological status, endotracheal intubation and mechanical ventilation were performed.

Hemodynamically, the patient was unstable on admission. Vasopressor therapy with norepinephrine was initiated at 0.026 µg/kg/min, along with fluid resuscitation. To maintain a target mean arterial blood pressure ≥65 mmHg, the norepinephrine dose was titrated to a maximum of 0.050 µg/kg/min. Fluid resuscitation was administered at 30 mL/kg over 3 h. Lactate levels decreased from 4.2 mmol/L to 3.4 mmol/L during therapy.

In addition to invasive arterial pressure monitoring, pulse wave parameters were continuously assessed using remote photoplethysmography. Under ongoing therapy, mean arterial pressure increased by 28%, (from 68 mmHg to 87 mmHg), while the mean perfusion index increased by 22% (from 0.67 a.u. to 0.82 a.u.). Dicrotic notch decreased by 11% (from 0.84 a.u. to 0.75 a.u.). SNR = 8.2 ± 2.1 (dB). Diastolic amplitude decreased by 27% (from 0.736 a.u. to 0.538 a.u.). The peripheral temperature was 36.1 °C, while axillary core temperature remained elevated at 38.5 °C.

**Figure 4:**
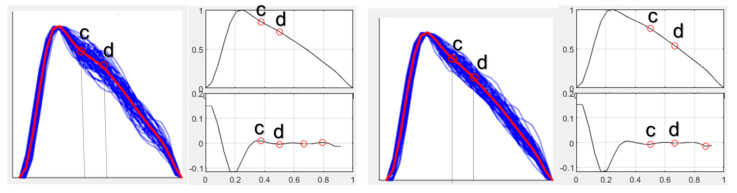
Pulse waveform analysis at baseline (**left**) and maximal norepinephrine dosage (**right**) for Case 1. Blue lines indicate single-period sPPG waveforms; the red line represents the ensemble-averaged waveform. The inflection points “c,d” are shown in the adjacent graph.

Pulse waveform during higher dose of norepinephrine (Figure 4):

Results show a strong positive correlation between NA and MAP (Pearson’s r = 0.843, *p* = 0.004) and between NA and PI (Pearson’s r = 0.752, *p* = 0.019) and a strong negative correlation between NA and c-wave (Pearson’s r = −0.747, *p* = 0.033); however, there was no correlation between NA and d-wave (Pearson’s r = 0.072, *p* = 0.865).

Case 2

A 62-year-old female patient was admitted to the hospital with complaints of vomiting and severe pain in the abdomen. Medical history included Crohn’s disease.

Abdominal computed tomography demonstrated the presence of free intraperitoneal fluid. The patient subsequently underwent an urgent laparotomy, which revealed an intestinal perforation. The perforation was identified and successfully repaired.

In the early postoperative period, the patient developed clinical signs of diffuse peritonitis, sepsis, and septic shock, requiring repeated exploratory laparotomy for source control. On admission to the intensive care unit, the patient was critically ill, with hemodynamic instability and severe metabolic acidosis. The initial SOFA score was 13. Laboratory studies demonstrated a markedly elevated lactate level of 7.8 mmol/L. Blood cultures grew *E. coli*.

Antimicrobial therapy was initiated with piperacillin–tazobactam in combination with fluconazole, considering the high risk of polymicrobial and fungal infection. The patient required endotracheal intubation and mechanical ventilation due to impaired neurological status.

Hemodynamically, the patient was unstable at admission. Vasopressor therapy with norepinephrine was initiated at 0.11 µg/kg/min, along with fluid resuscitation. To maintain a target mean arterial blood pressure ≥65 mmHg, the norepinephrine dose was titrated to a maximum of 0.27 µg/kg/min. Fluid resuscitation was administered at 30 mL/kg over 3 h. Lactate levels decreased from 7.8 mmol/L to 5.8 mmol/L during therapy.

In addition to invasive arterial pressure monitoring, pulse wave parameters were continuously assessed using remote photoplethysmography. Under ongoing therapy, mean arterial pressure increased by 40% (from 64 mmHg to 90 mmHg), while the mean perfusion index increased by 40% (from 0.10 a.u. to 0.14 a.u.). Dicrotic notch decreased by 5% (from 0.727 a.u. to 0.690 a.u.). Diastolic amplitude decreased by 10% (from 0.529 a.u. to 0.476 a.u.). SNR = 19.3 ± 1.3 (dB). The peripheral temperature was 31.7 °C, while axillary core temperature remained elevated at 38 °C.

**Figure 5:**
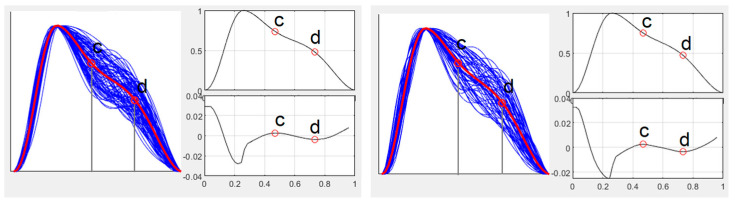
Pulse waveform analysis at baseline (**left**) and maximal norepinephrine dosage (**right**) for Case 2. Blue lines indicate single-period sPPG waveforms; the red line represents the ensemble-averaged waveform. The inflection points “c,d” are shown in the adjacent graph.

Pulse waveform during high dose of norepinephrine (Figure 5).

Results show a very strong positive correlation between norepinephrine NA and MAP (Pearson’s r = 0.997, *p* = 0.003) and between NA and PI (Pearson’s r = 0.990, *p* = 0.010), a strong negative correlation between NA and c-wave (Pearson’s r = −0.993, *p* = 0.007), and a negative correlation between NA and d-wave (Pearson’s r = −0.880, *p* = 0.004).

Case 3

A 55-year-old female patient was admitted to the hospital with complaints of progressive breathing difficulties and a high-grade fever of 38.5 °C. On admission, she was tachypneic and febrile, with clinical signs of respiratory distress.

Chest computed tomography (CT) revealed a large pleural effusion. The patient underwent video-assisted thoracoscopic surgery (VATS) for drainage of the effusion.

She was endotracheally intubated during the procedure and placed on mechanical ventilation under general anesthesia.

Following surgery, the patient was transferred to the intensive care unit (ICU) due to worsening hemodynamic instability. On ICU admission, she remained intubated and mechanically ventilated.

Hemodynamically, the patient was unstable, requiring vasopressor therapy with norepinephrine and fluid resuscitation at 30 mL/kg over 3 h to maintain adequate mean arterial pressure. Given the clinical suspicion of infection, broad-spectrum antibiotic therapy (piperacillin/tazobactam) was initiated with the working diagnosis of sepsis secondary to pleural empyema. Blood cultures, as well as cultures obtained from the pleural empyema, grew *E. coli*.

In addition to invasive arterial pressure monitoring, pulse wave parameters were continuously assessed using remote photoplethysmography. Under ongoing therapy, mean arterial pressure increased by 25% (from 71 mmHg to 89 mmHg), while the mean perfusion index increased by 15% (from 0.38 a.u. to 0.44 a.u.). Dicrotic notch decreased by 4% (from 0.925 a.u. to 0.896 a.u.). Diastolic amplitude decreased by 6% (from 0.856 a.u. to 0.806 a.u.). SNR = 18 ± 3.8 (dB). The peripheral temperature was 31.0 °C, while the axillary core temperature remained elevated at 39 °C.

**Figure 6:**
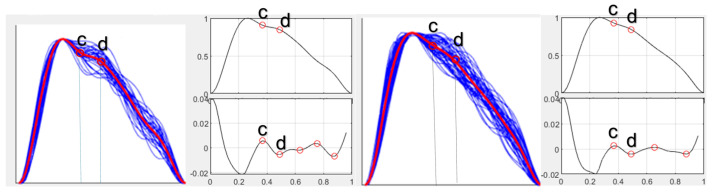
Pulse waveform analysis at baseline (**left**) and maximal norepinephrine dosage (**right**) for Case 3. Blue lines indicate single-period sPPG waveforms; the red line represents the ensemble-averaged waveform The inflection points “c,d” are shown in the adjacent graph.

Pulse waveform during high dose of norepinephrine (Figure 6):

Results show a very strong positive correlation between NA and MAP (Pearson’s r = 0.969, *p* = 0.0031) and between NA and PI (Pearson’s r = 0.966, *p* = 0.034), a strong negative correlation between NA and c-wave (Pearson’s r = −0.994, *p* = 0.006), and a negative correlation between NA and d-wave (Pearson’s r = −0.998, *p* = 0.002).

## 4. Discussion

This case series demonstrates that remote photoplethysmography (rPPG) is capable of detecting consistent pulse waveform alterations during vasopressor therapy in patients with septic shock. Across all three cases, increasing norepinephrine doses were associated with increases in mean arterial pressure (MAP) and perfusion index (PI), accompanied by reductions in dicrotic notch amplitude and diastolic wave amplitude. These findings indicate that rPPG is sensitive to the combined macrovascular and microvascular effects of vasopressor administration. As noted by Coutrot et al. [21], the perfusion index reflects not only pulsatile blood volume but also vascular tone, which is strongly modulated by autonomic (especially sympathetic) influences. This complexity means PI should be interpreted with caution, as it may not directly represent absolute peripheral blood flow under all physiological conditions. This physiological sensitivity may partly explain the variability observed in PI responses in our patients, particularly under vasoactive drug administration, and highlights a limitation of PI as a standalone surrogate of perfusion in critically ill settings.

### 4.1. Key Findings

The observed rise in PI and MAP aligns with the expected pharmacodynamic action of norepinephrine, which increases systemic vascular resistance and restores arterial pressure during distributive shock. Prior studies, such as He et al. (2022) [9], have shown that PI may increase in response to norepinephrine-mediated MAP augmentation, although this relationship is not always directly coupled to changes in cardiac output. In our cohort, the parallel increase in PI and MAP reflects improved peripheral pulsatility. However, it should not be interpreted as definitive evidence of microcirculatory recovery, given that vasoconstriction may redistribute blood flow rather than uniformly improve perfusion.

PI may reflect enhanced pulse pressure transmission from vasopressor-driven vasoconstriction rather than true improvement in nutritive microvascular perfusion. This limitation is particularly relevant in septic shock, where restoring MAP does not necessarily equate to restoring tissue-level flow.

Waveform morphology provides information that PI alone cannot. As described by Abushouk et al. [22], a reduction in c-wave amplitude is indicative of increased arterial stiffness and reduced vascular compliance, which is consistent with the α-adrenergic vasoconstrictive effects of norepinephrine observed in our patients. A consistent reduction in the dicrotic notch amplitude was observed in all patients during titration of vasopressor therapy. The dicrotic notch, which corresponds to aortic valve closure and is modulated by arterial compliance and peripheral wave reflections, is known to diminish under conditions of increased vascular tone or damping of the pulse wave. As described by Nirmalan and Dark (2014) [19], dicrotic notch morphology provides insight into systemic vascular resistance. In this context, the decreased notch amplitude likely reflects the heightened peripheral vasoconstriction induced by norepinephrine rather than impaired cardiac function. These waveform changes suggest that rPPG is capable of capturing alterations in arterial compliance that accompany catecholamine therapy. Furthermore, age-related increases in arterial stiffness have been shown to significantly affect PPG waveform morphology, including attenuation of the dicrotic notch and changes in pulse contour, which may influence baseline signal characteristics independent of acute hemodynamic interventions [23].

Similarly, the reduction in diastolic wave amplitude across all cases indicates changes in vascular tone and peripheral runoff rather than changes in cardiac filling [21,22]. This finding is consistent with previous work by Ter Horst et al. (2025) [24], who showed that sepsis progression is associated with altered PPG waveform morphology, including reduced amplitude components. Our observations demonstrate that rPPG-derived waveform features may remain sensitive even when traditional macrocirculatory parameters appear stable, underscoring the potential of rPPG for early detection of microvascular alterations or excessive vasoconstriction.

### 4.2. Interpretation and Comparisons

The broader literature supports the potential of PPG-based monitoring in critical care. Joachim et al. (2021) [15] demonstrated the feasibility of estimating MAP from PPG-derived features, while Kashchenko et al. (2023) [16] showed that PPG can reliably assess changes in tissue perfusion in intraoperative environments. Similarly, Middleton et al. (2011) [10] and Frey et al. (2008) [11] highlighted the value of PPG variability and waveform analysis in detecting physiological deterioration. The present findings extend this body of knowledge by demonstrating that rPPG—an entirely contactless form of PPG—can detect physiological changes during vasopressor titration in septic shock, a population characterized by profound microcirculatory dysregulation.

In interpreting our results, the correlations between norepinephrine doses and rPPG parameters (e.g., positive for PI and MAP, negative for c-wave and d-wave) suggest a dose-dependent response in waveform morphology. For instance, in Case 2, with the highest initial severity (SOFA 13), the strongest correlations were observed, potentially indicating greater sensitivity in more unstable patients. This aligns with the concept of hemodynamic coherence, where rPPG could serve as a bridge to detect mismatches between macro- and microcirculation.

### 4.3. Limitations

Despite these promising observations, several limitations must be acknowledged. The sample size is small, limiting statistical generalizability. Environmental conditions such as lighting and patient movement posed significant challenges to signal fidelity, consistent with known limitations of video-based photoplethysmography. Additionally, marked peripheral vasoconstriction—common in septic shock—may attenuate or distort waveform features, complicating interpretation. Despite the use of a controlled optical setup to minimize environmental interference, residual effects from ambient lighting variations in the ICU may still have influenced rPPG signal quality. In addition, although patients with diverse skin tones were included, we did not quantitatively evaluate potential differences in signal quality across skin tones, where increased light absorption may reduce signal amplitude. Therefore, the generalizability of the results across varying lighting conditions and skin pigmentation should be interpreted with caution. Future studies should systematically investigate these factors to further improve robustness. All patients were sedated and mechanically ventilated during data acquisition. Sedative and opioid agents are known to modulate autonomic tone and peripheral vasoreactivity, potentially confounding waveform interpretation. Although sedation regimens remained stable during observation periods, their effects on rPPG-derived parameters cannot be fully excluded. Future studies should validate rPPG-derived microcirculatory indices against established reference techniques, such as sublingual video microscopy and near-infrared spectroscopy (NIRS), to better determine their sensitivity and specificity for assessing true microvascular perfusion and hemodynamic coherence in critically ill patients. Findings may not generalize to diverse demographics; future studies should include males and broader age ranges. Nevertheless, these limitations reflect real-world constraints in the intensive care environment and highlight areas where technical refinement is essential.

### 4.4. Future Directions

To build on this feasibility study, larger controlled trials are recommended, incorporating control groups (e.g., non-septic patients or alternative vasopressors) and diverse populations to assess generalizability. Integration of artificial intelligence for real-time artifact correction could enhance rPPG robustness in ICU settings. Additionally, comparing rPPG with gold-standard microcirculatory tools like NIRS or video microscopy would validate its clinical utility. Exploring multi-wavelength rPPG or combination with other non-invasive modalities (e.g., wearables) could further elucidate its role in monitoring hemodynamic coherence and guiding vasopressor titration in septic shock.

## 5. Conclusions

In conclusion, this case series demonstrates the feasibility of using remote photoplethysmography (rPPG) to detect pulse waveform changes during norepinephrine therapy in patients with septic shock. Waveform-derived features may provide complementary information on vascular dynamics beyond conventional macrocirculatory parameters. However, given the small sample size and lack of a control group, the observed associations cannot be interpreted as causal. Larger controlled studies are required to validate these findings and clarify the potential clinical role of rPPG in critical care monitoring.

## Figures and Tables

**Table 1:** Baseline characteristics, diagnoses and medication requirements of the three patients included in this study.

Patient Nr.	Age, Years	Height, cm	Weight, kg	SOFA Score	Diagnosis and Bacterial Cultures	Initial Norepinephrine Dose (µg/kg/min)	Midazolam (mg/kg/min)	Fentanyl (µg/kg/min)
1	49	168	85	8	Pneumonia, ARDS; *Legionella*	0.026	1.26	0.03
2	62	172	67	13	Peritonitis; Escherichia (*E. coli*)	0.11	1.02	0.03
3	55	169	80	9	Pleural empyema, pneumonia; *E. coli*	0.02	1.50	0.05

## Data Availability

The original contributions presented in this study are included in the article’s material; further inquiries can be directed to the corresponding author(s).

## Abbreviations

The following abbreviations are used in this manuscript:

ARDS	Acute Respiratory Distress Syndrome
CWL	Center Wavelength
FWHM	Full Width at Half Maximum
a.u.	Arbitrary units
d-wave	Diastolic wave amplitude
CT	Computed Tomography
*E. coli*	*Escherichia coli*
MAP	Mean arterial pressure
ICU	Intensive Care Unit
NA	Norepinephrine
PI	Perfusion Index
PPG	Photoplethysmography
rPPG	Remote Photoplethysmography
SOFA	Sequential Organ Failure Assessment
SPPG	Subspace Photoplethysmography
VATS	Video-Assisted Thoracoscopic Surgery

## References

[1] Evans L., Rhodes A., Alhazzani W., Antonelli M., Coopersmith C.M., French C., Machado F.R., Mcintyre L., Ostermann M., Prescott H.C. (2021). Surviving Sepsis Campaign: International Guidelines for Management of Sepsis and Septic Shock 2021. Crit. Care Med..

[2] Singer M., Deutschman C.S., Seymour C.W., Shankar-Hari M., Annane D., Bauer M., Bellomo R., Bernard G.R., Chiche J.-D., Coopersmith C.M. (2016). The Third International Consensus Definitions for Sepsis and Septic Shock (Sepsis-3). JAMA.

[3] Charlton M., Sims M., Coats T., Thompson J.P. (2017). The microcirculation and its measurement in sepsis. J. Intensive Care Soc..

[4] Xantus G., Allen P., Kanizsai P. (2021). Blind spot in sepsis management—Tissue level changes in microcirculation. Physiol. Int..

[5] Ospina-Tascon G.A., Madrinan-Navia H. (2015). Should microcirculation monitoring be used to guide fluid resuscitation in severe sepsis and septic shock?. Rev. Bras. Ter. Intensiv..

[6] De Backer D. (2019). Is microcirculatory assessment ready for regular use in clinical practice?. Curr. Opin. Crit. Care.

[7] Allen J. (2007). Photoplethysmography and its application in clinical physiological measurement. Physiol. Meas..

[8] Magro G. (1952). Clinical applications of photoplethysmography. Arch. Mal. Coeur Vaiss..

[9] He H.-W., Liu W.-L., Zhou X., Long Y., Liu D.-W. (2020). Effect of mean arterial pressure change by norepinephrine on peripheral perfusion index in septic shock patients after early resuscitation. Chin. Med. J..

[10] Middleton P.M., Tang C.H.H., Chan G.S.H., Bishop S., Savkin A.V., Lovell N.H. (2011). Peripheral photoplethysmography variability analysis of sepsis patients. Med Biol. Eng. Comput..

[11] Frey B., Waldvogel K., Balmer C. (2008). Clinical applications of photoplethysmography in paediatric intensive care. Intensive Care Med..

[12] Elgendi M., Jost E., Alian A., Fletcher R.R., Bomberg H., Eichenberger U., Menon C. (2024). Photoplethysmography Features Correlated with Blood Pressure Changes. Diagnostics.

[13] Elgendi M., Haugg F., Fletcher R.R., Allen J., Shin H., Alian A., Menon C. (2024). Recommendations for evaluating photoplethysmography-based algorithms for blood pressure assessment. Commun. Med..

[14] Tusman G., Acosta C.M., Pulletz S., Böhm S.H., Scandurra A., Arca J.M., Madorno M., Sipmann F.S. (2019). Photoplethysmographic characterization of vascular tone mediated changes in arterial pressure: An observational study. J. Clin. Monit. Comput..

[15] Joachim J., Coutrot M., Millasseau S., Matéo J., Mebazaa A., Gayat E., Vallée F. (2021). Real-time estimation of mean arterial blood pressure based on photoplethysmography dicrotic notch and perfusion index. A pilot study. J. Clin. Monit. Comput..

[16] Kashchenko V.A., Lodygin A.V., Krasnoselsky K.Y., Zaytsev V.V., Kamshilin A.A. (2023). Intra-abdominal laparoscopic assessment of organs perfusion using imaging photoplethysmography. Surg. Endosc..

[17] Adda I., Lai C., Teboul J.-L., Guerin L., Gavelli F., Monnet X. (2021). Norepinephrine potentiates the efficacy of volume expansion on mean systemic pressure in septic shock. Crit Care.

[18] Smith M.D., Maani C.V. (2025). Norepinephrine. StatPearls.

[19] Nirmalan M., Dark P.M. (2014). Broader applications of arterial pressure wave form analysis. Contin. Educ. Anaesth. Crit. Care Pain.

[20] Rubins U., Marcinkevics Z., Grabovskis A. Neural Network-Based Feature Prediction Using Remote Photoplethysmogram Waveform Analysis. Proceedings of the 2024 IEEE 14th International Conference on Nanomaterials: Application & Properties (NAP 2024).

[21] Coutrot M., Dudoignon E., Joachim J., Gayat E., Vallée F., Dépret F. (2021). Perfusion index: Physical principles, physiological meanings and clinical implications in anaesthesia and critical care. Anaesth. Crit. Care Pain Med..

[22] Abushouk A., Kansara T., Abdelfattah O., Badwan O., Hariri E., Chaudhury P., Kapadia S.R. (2023). The Dicrotic Notch: Mechanisms, Characteristics, and Clinical Correlations. Curr. Cardiol. Rep..

[23] Charlton P.H., Paliakaitė B., Pilt K., Bachler M., Zanelli S., Kulin D., Allen J., Hallab M., Bianchini E., Mayer C.C. (2022). Assessing hemodynamics from the photoplethysmogram to gain insights into vascular age: A review from VascAgeNet. Am. J. Physiol. Heart Circ. Physiol..

[24] Ter Horst S., van Wijk R.J., Schoonhoven A.D., de Lange A., ter Maaten J.C., Bouma H.R. (2025). Pulse oximetry beyond oxygen saturation: Early waveform characteristics in sepsis patients with adverse outcomes—A proof-of-concept study. J. Crit. Care.

